# Neuro-emotions based on electroencephalograph response to different color *Ardisia mamillata Hance* plants in elderly people with and without cognitive impairment

**DOI:** 10.3389/fpubh.2022.955393

**Published:** 2022-10-31

**Authors:** Juan Du, Xiaomei Chen, Xi Li, Yuanzhi Pan, Erkang Fu, Yumei Huang, Chunyan Zhu, Mingyan Jiang, Ahmad Hassan, Dingru Wu, Jun Ma, Guangsheng Yuan

**Affiliations:** ^1^College of Landscape Architecture, Sichuan Agricultural University, Chengdu, China; ^2^Yijia Apartment, Chengdu, China; ^3^Maize Research Institute, Sichuan Agricultural University, Chengdu, China

**Keywords:** EEG, plant color, *Ardisia mamillata Hance*, elderly, cognitive impairment, neuro-emotion

## Abstract

**Background:**

Given the aging population, the quality of mental health of elderly people deserves special attention. The aims of this study were (1) to assess the difference of neuro-emotion based on EEG from the cognitively impaired elderly (CNE) and the cognitively normal elderly (CIE) participants viewing different color *Ardisia mamillata Hance* and (2) to determine which color *Ardisia mamillata Hance* has greater benefits for boosting their neuro-emotions.

**Methods:**

The cognitive function of the participants was judged by using the revised Chinese version of the Mini-Mental State Examination (MMSE) scale combined with the daily cognitive performance of the participants, and the participants were divided into the cognitive normal elderly (CNE) and the cognitive impairment elderly (CIE). A total of 10 CNE volunteers and 10 CIE volunteers were recruited as participants for this study. For this study, two varieties of *Ardisia mamillata Hance*, green tiger tongue (GTT) with green leaves and red tiger tongue (RTT) with reddish brown leaves, were observed as plant materials. In total, six emotional indexes, including stress, engagement, interest, excitement, focus, and relaxation, were then measured by electroencephalography (EEG).

**Results:**

RTT had the most positive effect on EEG neuro-emotion in the CNE group, with significant reductions in stress, engagement, and focus in the RTT test, while the combination of GTT+RTT had a positive effect on EEG neuro-emotions in the CIE group, with significant reductions in engagement and focus in the GTT+RTT test. No statistically significant differences were found for the interest, excitement, and relaxation index in the CNE and CIE participants in all tests.

**Conclusion:**

Significant reductions were observed in stress, engagement, and focus values of the CIE participants in the RTT test, which indicated that the CNE participants were more relaxed. RTT is a reddish brown and warm color plant, so the CNE individuals should always have the warm color plants indoors or outdoors, which could help boost their neuro-emotions. Significant reductions were observed in engagement and focus values of the CIE participants in the GTT+RTT test, which indicated that the CIE participants were more relaxed. The combination of GTT+RTT test shows the combination cold and warm color plants; therefore, the CIE individuals should always have a combination of cool and warm color plants indoors or outdoors, which could help boost their EEG neuro-emotions.

## Introduction

With the aging population, people aged 60 years and older are expected to exceed 20% of the total population by 2050 (1). Currently, China has the largest population of older people in the world (2). According to a 2019 survey, people over 65 years old accounted for 12.6% of the total population in China (3). Based on a recent report from the United Nations, over a third of Chinese people will be over 60 years old by 2050 (4). Physical and mental health problems such as anxiety, insomnia, depression, loneliness, and cognitive impairment are easily induced among older people due to a decline in physical function and social isolation. Thus, in an increasingly aging society, the health of older people is a worthwhile research topic.

### Plants and human health

Recently, much progress has been made toward understanding the relationship between plants and human health. A large number of scholars have reported that indoor plants have a positive effect on human health (5–8). For instance, one report showed that having yellow chrysanthemums on the table significantly increased meal times, food consumption, and vocalizations in patients with a psychiatric disorder (9). Fjeld et al. reported that total scores for fatigue, dry throat cough symptoms, and dry skin symptoms in participants decreased 21% after interacting with plants (10). The same team also found that discomfort symptoms in subjects who interacted with plants decreased by 21% to 25% compared with those who did not (11). Another study found that people interpreted the presence of plants as an air cleaner, and this made them feel more relaxed (12). Park et al. reported that patients in rooms with plants had greater pain tolerance and lower self-ratings of pain intensity than controls in rooms without plants (13). Dijkstra et al. showed that patients exposed to indoor plants had lower stress than the control group. Taken together, these findings suggest that indoor plants can reduce stress by increasing the attractiveness of rooms (14). As we all know, plants are an important part of nature, with functions such as ecology, beautification, and healthcare, and have a positive effect on the health life of the elderly too (15). Numerous studies have shown that exposure to green environments can lead to higher levels of well-being in older adults and lessen the intrusion of anxiety and stress (16–18). Meil X and others found that gardening activities that are in close contact with plants through rose pruning, cuttings, potted plant making, etc., can help the elderly to reduce loneliness and increase more positive and optimistic emotions about life, thus reacting to the body and achieving physical and mental improvement and, at the same time, good benefits (19). Zhang et al. studied the outdoor activity space of the elderly and found that vibrant flowers and plants can eliminate the negative emotions from them and make them experience the joy of life (20). Xiaox et al. researched the effects of bamboo on the physical and mental health of the elderly and found that bamboo can promote the mental health of the elderly. The stress value and attention value of the elderly were significantly reduced, and the relaxation value was significantly increased (21). Hassan et al. reported that the elderly had lower blood pressure, felt more comfortable and relaxed, and had lower anxiety levels after the plant task (22). Plants always have been utilized to release stress and depression and improve mood and psychological stability (23, 24).

### Plant color and human emotion

Although humans perceive environmental information through their five senses, 70% of such information comes from vision (25). It is known that color is closely related to people's emotion. Many research efforts have focused on how color affects emotional response, which is termed color emotion research (26). There are two categories of research in this field: (i) experimental aesthetics of color or color preference, which involves dimensions of color evaluation, such as comfort or discomfort, and good or bad (27–29), and (ii) descriptive dimensions of color, such as warm or cold, bright or dark, and deep or light (30–37). Many reports have shown that plant color can have both positive physiological and psychological effects. Purple and green plant landscapes were shown to be more effective than yellow, red, and white plant landscapes in relieving anxiety, relaxing the body, and improving mood (37). Sadek et al. found that green plants were preferred by college students over green–red and green–white plants. In addition, green plants had a greater effect than other color plants in relaxing bodies and had a sedative effect on cerebral blood flow in sensory areas of the brain (38). Jialin et al. reported that red and purple flowers were the best choice for increasing happiness among subjects (20–35 years old), that yellow or red flowers have a better effect on stress relief, that white and blue flowers have the strongest effect on soothing tension, and that blue or purple flowers have the best effect on recovery of attention and cognitive levels (39). Another report found that a little bit of greenery in a limited space could deeply affect relaxation (40).

### Study of neuro-emotions based on electroencephalograph

In recent years, many scholars have explored people's emotional changes by studying their brain response to plants: one of the ways is to test the neuro-emotion of subjects in the environment with plants directly by electroencephalography (EEG). This method is the most direct, and the value of various emotions (e.g., stress, engagement, interest, excitement, focus, and relaxation) can be directly read by software matched with the EEG. EEG has been used as clinical equipment to record neuro-emotions (41–44). For example, Lin et al. (45) reported that the neuro-emotional parameters of walking and sitting groups in an urban green space can be dynamically measured using EEG headphones. Their results showed that the valence and meditation values of the walking group were higher than those of the sitting group, indicating that walking is an efficient way to reduce stress. However, the focus value of the sitting group was higher than that of the walking group, suggesting that sitting has more benefit on the recovery of attention. Du et al. (46) used EEG to assess neuro-emotions in elderly people with normal and impaired cognition during flower arrangement. They found that focus value decreased in normal elderly participants, while engagement and interest values increased in elderly people with cognitive impairment. Zeng et al. (47) found that vegetation density (VD) and integrated sound environment (ISE) had significant effects on the neuro-emotions of college students, with higher VD likely leading to the expression of excitatory neuro-emotions. In the study by Neale et al. (44) used five neuro-emotional parameters provided by EEG to compare the walking status of the elderly in three different urban environments, and they found that compared with the busy and quiet urban environment, walking in urban green spaces has higher levels of “engagement” and better restorative effects in the elderly. Rodríguez et al. (48) used a portable EEG device to study changes in brain activity during negative emotion induction in a virtual park environment, compared the brain activity of participants before and after emotion induction, and found that both evoked their negative emotions (sadness), which supports this approach as possible tools to test emotions. Roe et al. used EEG to assess the restoration potential of viewing natural environments in a laboratory setting and showed that natural landscapes have higher “meditation” and lower “excitement” than urban environments, with better restoration benefits (49), which confirms the reliability of using the EEG instrument to study neuro-emotion. In this study, this method was used to measure the EEG neuro-emotion of participants.

Despite recent progress in exploring the effects of different plants on elderly's health, there is no research on differences in neuro-emotion between the cognitively normal elderly (CNE) people and cognitively impaired elderly (CIE) people viewing different colors of plants. For this study, two different color plants were selected as the visual stimuli for elderly participants in this study, namely, the green tiger tongue (GTT) with green leaves and red tiger tongue (RTT) with reddish brown leaves, which are variants of *Ardisia mamillata Hance*. The aims of this study were (1) to assess the difference of EEG neuro-emotion in the CNE and CIE participants viewing different color *Ardisia mamillata Hance*, and (2) to determine which color *Ardisia mamillata Hance* has greater benefits for boosting their neuro-emotions. The findings of the present study could provide guidance for different groups of elderly people to select suitable colors of plants to keep in homes or outdoors for good neuro-emotions.

### Participants

Elderly participants were recruited on a voluntary basis from a nursing house in Wenjiang county, Chengdu city, China. The nursing house has more than 40 elders older than 60 years, which facilitated the recruitment of participants. People older than 60 years without severe cognitive impairment were eligible to participate. In this experiment, to determine the emotional stability of the participants, a revised Chinese version of the Mini-Mental State Examination (MMSE) scale was used to screen the cognitive function of the participants (50). In this scale, the full score is 30; considering the educational background of the participants, the score of cognitive impairment was given as follows: ≤17 points for no education, ≤20 points for primary school education, and ≤24 points for secondary or higher education. The degree of cognitive impairment (DMI) was as follows: mild ≥21, moderate10–20, and severe ≤9. In addition to the MMSE scores of the participants, it was also necessary to determine whether the participants had cognitive impairment, we combined the MMSE with the daily cognitive ability evaluation with the help of nursing home staff. As a result of sample screening, only 20 old people aged 80–90 years were eligible to participate in the experiment, including 10 cognitively normal elderly (CNE) participants and 10 cognitively impaired elderly participants (CIE). The DMI of the CIE individuals was moderate (Table 1). All the participants provided written informed consent. The experiment was carried out from 15 April to 18 April 2021 at 9:30–11:30 a.m. and 2:30–5:00 p.m. in a quiet indoor room measuring 3.6 meters wide and 6 meters long in the nursing house. The illumination, humidity, and temperature of the room were maintained at 500 lux, 50–55%, and 22–25°C, respectively.

**Table 1:** Participants.

	**CNE**				**CIE**				
**n**	**Age**	**Gender**	**Education**	**MMSE score**	**Age**	**Gender**	**Education**	**MMSE score**	**DMI**
1	82	M	No	20	90	M	Primary	14	Moderate
3	80	F	Primary	21	89	F	Secondary	15	Moderate
4	83	M	Secondary	22	88	F	No	13	Moderate
5	86	M	No	19	81	F	Secondary	20	Moderate
6	82	F	No	18	84	F	Primary	18	Moderate
7	80	F	Primary	24	83	M	Primary	19	Moderate
8	85	F	Primary	27	82	F	No	12	Moderate
9	81	F	Secondary	26	81	F	Primary	17	Moderate
10	83	M	Primary	23	80	M	Secondary	17	Moderate

### Plant materials

**Figure 1:**
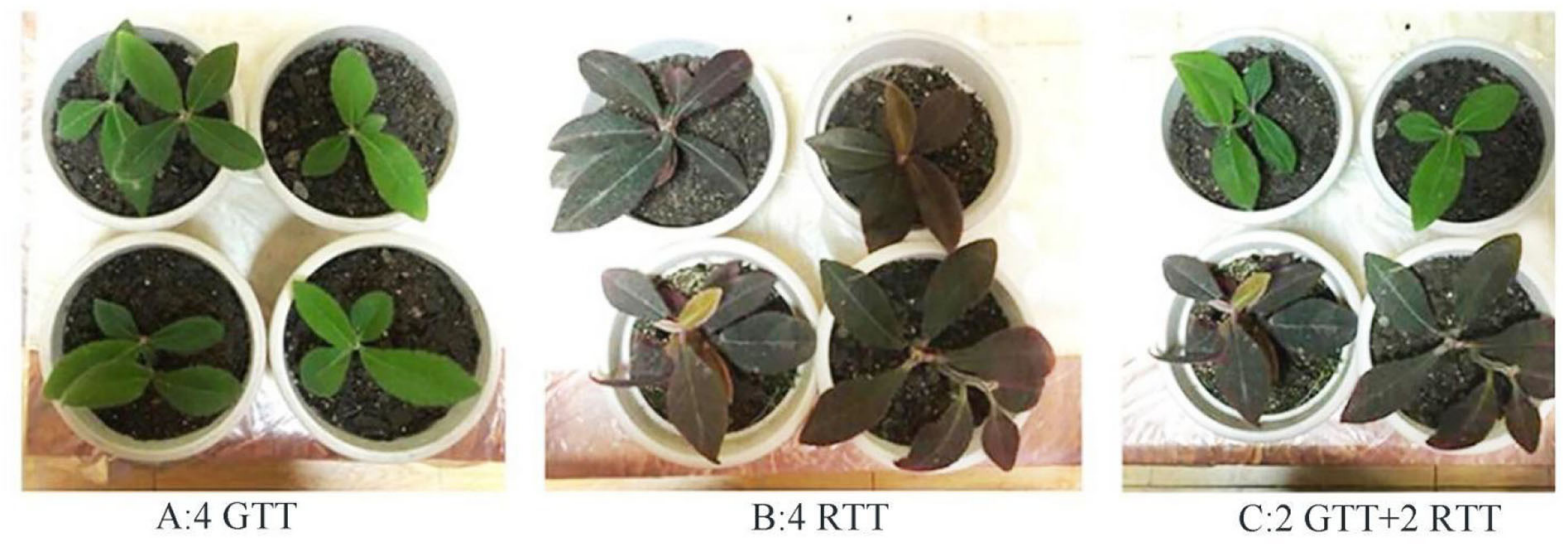
Plant materials.

*Ardisia mamillata Hance* is an ornamental plant used in the study for both indoor and outdoor viewing, and it has two different colored varieties, namely, green tiger tongue (RTT) (Figure 1A) and red tiger tongue (GTT) (Figure 1B). GTT has a green leaf color, while RTT has a reddish brown leaf color. So, they were chosen to be used in this study. A total of 4GTT, 4RTT, and 2GTT+ 2RTT (2GTT were from 4GTT, 2RTT were from 4RTT) were used as the observation materials in this study, as shown in Figure 1. Before the experiment, the plant materials should be cleaned for use.

### EEG measurements

**Figure 2:**
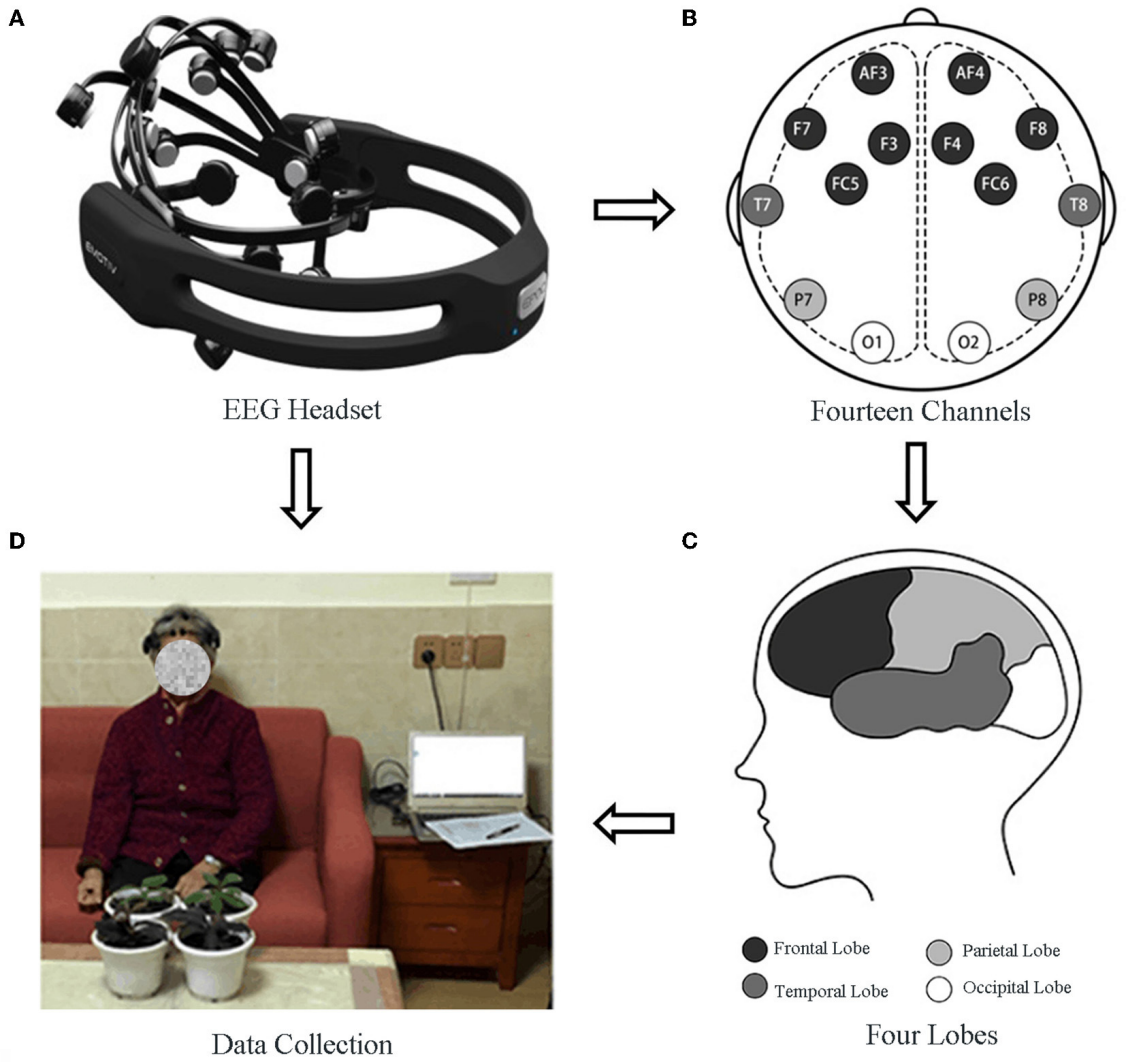
EGG Data collection.

Participants' brain wave signals were collected using emotional EPOC + EEG headphones (Figure 2A). The four cerebral lobes (frontal, occipital, parietal, and temporal lobes) were measured using 14 electrodes (O1, O2, AF3, F3, AF4, F4, FC5, FC6, F7, P7, T7, F8, T8, and P8) of the headphones (Figures 2B,C). Among them, the frontal lobe (F3, F4, AF3, AF4) mainly reflects emotion, language, cognition, and behavior. The temporal lobe (T7, T8) mainly controls language processing and audio-visual memory, the parietal lobe (P7, P8) mainly controls reception and sensory connection, and the occipital lobe (O1, O2) mainly reflects visual information in the brain. The collected EEG signals were inputted into a computer through a Bluetooth device (Figure 2D). Overall, six emotional values (stress, excitement, interest, focus, relaxation, and engagement) were recorded each minute by Emotiv Pro software to reflect brain activity and emotional change, and these emotion values can be read directly from the software.

### Procedure

**Figure 3:**
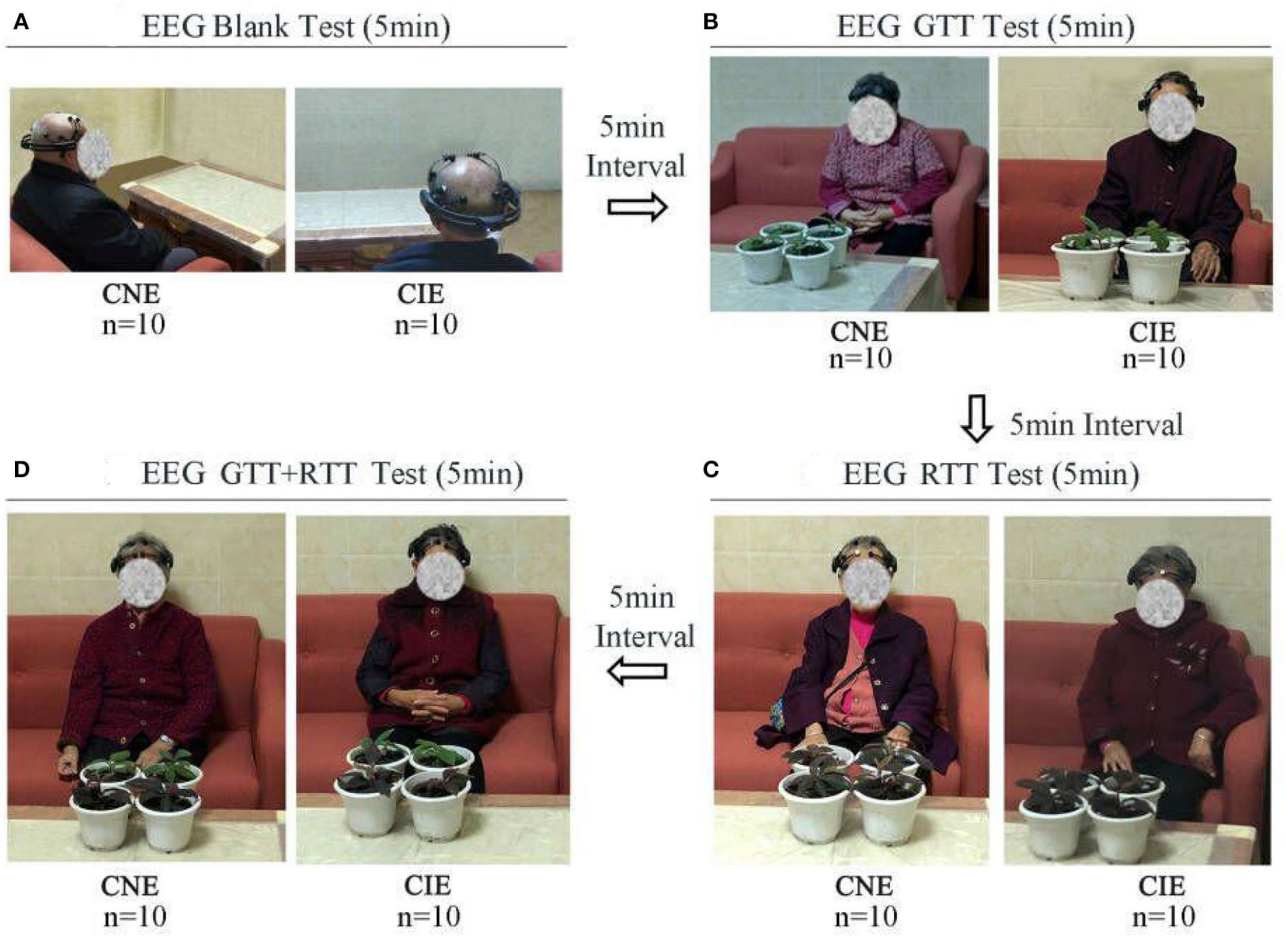
Experimental procedure.

Before the experiment, the procedure was explained to all participants, and they were required to sit calmly for 5 min before starting. When the experiment began, the participants first underwent a blank test, where no plants were placed on the table (Figure 3A). Next, the three types of plants (4GTT, 4RTT, and 2GTT+2RTT) were placed on the table for participants' observation (Figures 3B–D). The plants were placed in a position where the participants could see them clearly and easily. For each test (EEG GTT test, EEG RTT test, and EEG GTT+RTT test), emotional values were collected once per minute for a total of five times in a 5-min test, and the average of the five test scores were used as the final test value. The duration of each test was 5 min. The interval between each test was also 5 min (Figures 3A–D).

### Statistical analysis

Statistical analysis of six emotion values was performed by using SPSS 24.0 (SPSS, Armonk, NY, USA) software. The paired *t*-test was used to analyze differences between the two groups when the data showed normality and homoscedasticity; otherwise, the Wilcoxon signed-rank test was used to analyze the differences of both the groups.

## Results

### Differences in neuro-emotion value

**Figure 4:**
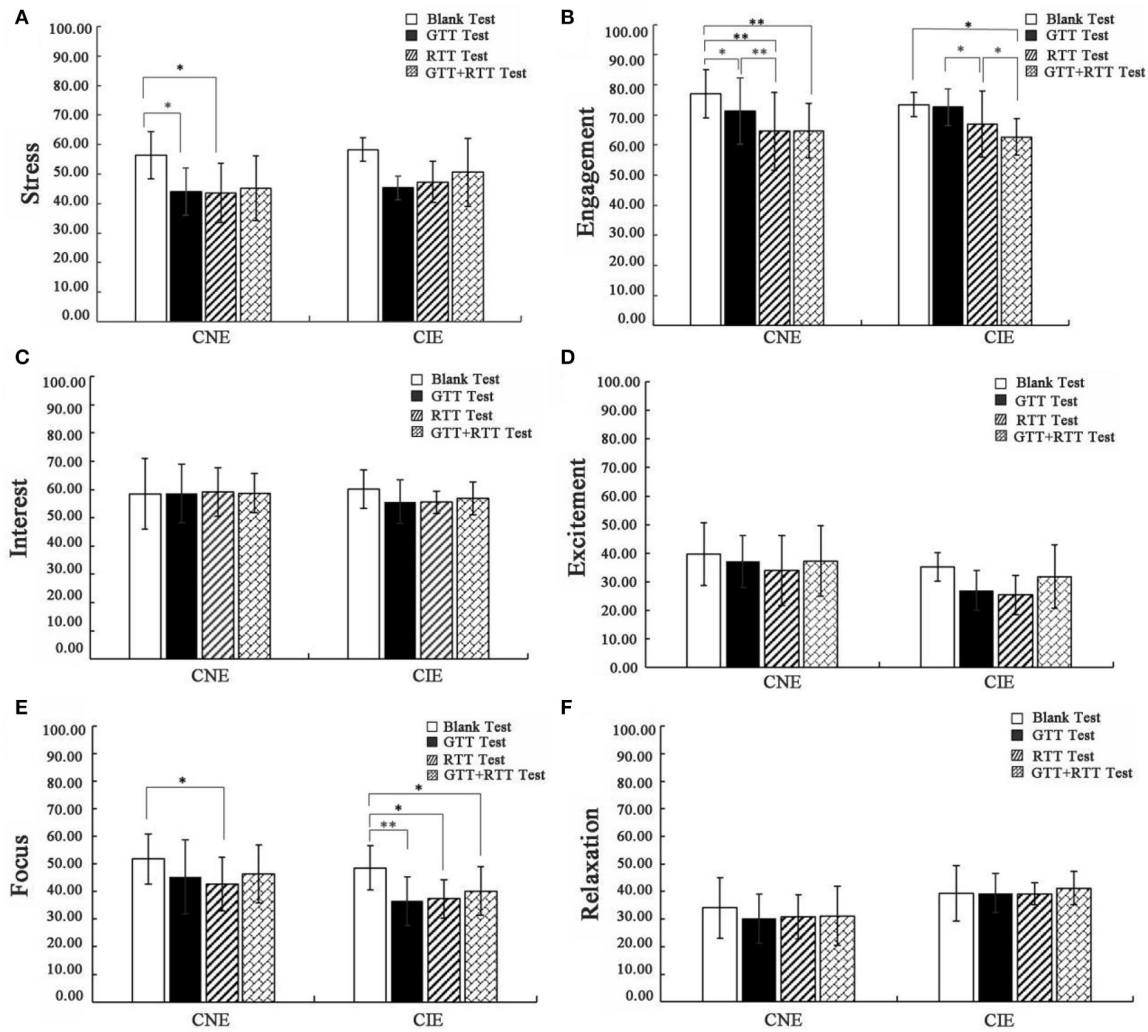
Significance of difference analysis between tests (^**^sig. <0.01, ^*^sig, <0.05). **(A)** Stress. **(B)** Engagement. **(C)** Interest. **(D)** Excitement. **(E)** Focus. **(F)** Relaxtion.

For the stress value, in four tests (viz., blank test, GTT test, RTT test, and GTT+RTT test) conducted for the CNE participants, the stress value decreased, with significant decreases in the GTT test (t = 2.49, *p* <0.05) and RTT test (*t* = 2.82, *p* <0.05) (Figure 4A). while for the CIE participants, the stress value decreased (Figure 4A). These results showed that stress levels in both the groups were reduced in the presence of plants compared with no plants; however, compared with the CIE participants, the presence of plants has a more obvious effect on the stress reduction of the CNE participants.

For the engagement value, in the four tests for the CNE participants, the engagement value decreased significantly (Figure 4B), with the lowest value in the RTT test (t = 5.13, *p* < 0.01) and GTT+RTT test (t = 5.11, *p* < 0.01), while in the four tests for the CIE participants, the engagement value also decreased significantly (Figure 4B), with the lowest value in the GTT+RTT test (t = 2.65, *p* < 0.05). These results showed that participants in both the groups had the lowest levels of engagement in the GTT+RTT test.

For the focus value, in the four tests for the CNE participants, a significant decrease was observed in the RTT test (t = 2.30, *p* < 0.05) (Figure 4E), while in four tests for the CIE participants, the value of focus decreased significantly (Figure 4E), with the lowest value appearing in the GTT test (t = 3.76, *p* < 0.01). These results indicated that for the CNE participants, RTT has the best effect on reducing their focus value, while for the CIE participants, GTT has the best effect on reducing their focus value.

Regarding the excitement and relaxation values of the CNE and CIE participants in all tests, there was no statistically significant differences (Figures 4C,D,F).

Overall, RTT had the most positive effect on EEG neuro-emotion in the CNE group, with significant reductions in stress, engagement, and focus in the RTT test, while GTT+RTT had the positive effect on EEG neuro-emotions in the CIE group, with significant reductions in engagement and focus values in the GTT+RTT test.

## Discussion

This study presented differences in EEG-based neuro-emotion responses to viewing GTT and RTT plants in the CNE and CIE groups.

First, RTT had the most positive effect on EEG neuro-emotion in the CNE group, with significant reductions in stress, engagement, and focus in the RTT test. The decrease in stress indicated that the CIE participants were in a more relaxed state. As engagement could reflect the degree of immersion, investment, or attraction (45), the decrease of engagement value suggested that the CNE participants were also in a more relaxed state. Focus was a high state of arousal, reflecting high attention (45). Therefore, the decrease in focus was a good sign in this study, indicating that the CNE participants were more relaxed with the RTT. Therefore, we could conclude that in this study, RTT was more beneficial to the EEG neuro-emotion of the CNE participants than the GTT and GTT+RTT. RTT induced a more relaxed state in the CNE participants. RTT is a kind of reddish brown plant, which has a warm tone. Therefore, we could think that warm-toned plants were more beneficial to the EEG neuro-emotion of the CNE participants in this experiment. This finding was inconsistent with some previous studies. For instance, Li et al. (37) reported that green and purple plantscapes were more effective in relaxing the body, reducing anxiety, and improving mood in university students, relative to red, yellow, and white plantscapes. Sadek et al. (38) also found that university students preferred green to green–red and green–white plants. These reports suggested that green plants can make people more relaxed than red, red–green, white–green, yellow, and white plants. In the present study, the CNE group preferred reddish brown (RTT) to green (GTT) plants. This interesting phenomenon could be due to the difference in age between the study participants. In contrast to the aforementioned studies involving college students and young adults, the subjects in our study were older people, and the two groups may have different responses to color. Jung et al. (51) found that elderly people prefer warm colors to cold colors. A color characteristics analysis showed that yellow and yellow red colors were preferred by 70% of people at a senior citizen center, which coincides with elders' preference for warm colors (52). Due to the gradual decline of physiological function, the color preference of elderly people may be different from that of young people. Thus, housing facilities for older people should consider the unique color preferences of the elderly, such as warm and bright colors like red and yellow (53). As reddish brown (RTT) is a warm color, it may have made older people feel more relaxed. This result supports that warm color plants can help elderly people feel more relaxed, but this finding might not apply to young people. Given these findings, we suggest that CNE individuals should always have warm color plants indoors or outdoors, which could help boost their emotions.

Second, GTT+RTT had a positive effect on EEG neuro-emotion in the CIE group, with significant reductions in engagement and focus values in the GTT+RTT test. As mentioned earlier, “engagement” can reflect the degree of immersion, investment, or attraction. “Focus” is a high state of arousal, reflecting high attention (45). Therefore, the decrease in these two emotion values indicated that the CIE participants were in a more relaxed state with GTT+RTT. GTT is green and cold color plant, while RTT is reddish brown and warm color plant; therefore, in this study, the combination of cool and warm colors was more beneficial to the EEG neuro-emotion of the CIE participants. This is where the CIE participants differ from the CNE participants. Colorful plants were better for the mood of the CIE participants. Wang et al. (54) reported that chromatic and achromatic colors led to different relaxation responses. Colorful plants can stimulate vison and improve sleep quality in CIE people and reduce restless behavior (55). The conclusions of these previous studies are similar to those of this study. As we all know that the gradual loss of cognitive ability and mental stability in the CIE group may produce abnormal behaviors that pose a danger to society (56), and relaxed emotion state could reduce the occurrence of destructive behaviors from the CIE individuals (57). Given these findings, we suggest that the CIE individuals should always have the combination of cool and warm color plants indoors or outdoors, which could help boost their EEG neuro-emotions.

Last, there are some limitations to this study. First, the sample size is too small. In future research, we hope to expand the number of subjects to collect more data. Second, in this study, it is not enough to only use the MMSE score and daily performance of participants to diagnose whether they have cognitive dysfunction, and future studies need to adopt more rigorous and scientific diagnostic methods. Also, the use of only two plant colors may be too few. In follow-up studies, more plant colors should be considered to explore elderly people's responses to multicolor plants.

## Conclusions

In the present study, we used EEG to demonstrate significant differences in neuro-emotion responses to different color *Ardisia mamillata Hance* between the CNE and CIE participants. RTT had the most positive effect on EEG neuro-emotion in the CNE group, with significant reductions in stress, engagement, and focus in the RTT test, indicating that the CNE participants were more relaxed with RTT, which is reddish brown and a warm color plant, which implies that the CNE individuals should always have warm color plants indoors or outdoors, which could help boost their neuro-emotions. GTT+RTT had the positive effect on EEG neuro-emotions in the CIE group, with significant reductions in engagement and focus in the GTT+RTT test, indicating that the CIE participants were more relaxed. In this study, the combination of cool and warm colors plants were more beneficial to the EEG neuro-emotion of the CIE participants. Hence, CIE individuals should always have the combination of cool and warm color plants indoors or outdoors, which could help boost their EEG neuro-emotions.

## Data availability statement

The original contributions presented in the study are included in the article/supplementary material, further inquiries can be directed to the corresponding authors.

## Ethics statement

The studies involving human participants were reviewed and approved by the College of Sichuan Agricultural University, China. The patients/participants provided their written informed consent to participate in this study.

## Author contributions

JD: experimental design and manuscript preparation. XC: manuscript preparation. XL and GY: experimental design and editing. YP: experimental design and review. EF and DW: experimental design. YH: data analysis. CZ: review and editing. MJ: data analysis. AH: review. JM: experimental progress and review. All authors contributed to the article and approved the submitted version.

## Conflict of interest

The authors declare that the research was conducted in the absence of any commercial or financial relationships that could be construed as a potential conflict of interest.

## Publisher's note

All claims expressed in this article are solely those of the authors and do not necessarily represent those of their affiliated organizations, or those of the publisher, the editors and the reviewers. Any product that may be evaluated in this article, or claim that may be made by its manufacturer, is not guaranteed or endorsed by the publisher.
